# Cardiac calcified amorphous tumor in the right atrium: a rare cardiac neoplasm

**DOI:** 10.1186/s13019-025-03501-y

**Published:** 2025-07-09

**Authors:** Wentao Fu, Kun Liu, Yan Zhang, Jing Wang, Wan Cai, Yaoyao Wu, Hao Chi, Wen Ge

**Affiliations:** 1 Department of Cardiothoracic Surgery, Shuguang Hospital, Shanghai University of Traditional Chinese Medicine, Shanghai, China; 2 Department of Pathology, Shuguang Hospital, Shanghai University of Traditional Chinese Medicine, Shanghai, China; 3 Ultrasound Department, Shuguang Hospital, Shanghai University of Traditional Chinese Medicine, Shanghai, China

**Keywords:** Non-neoplastic mass, Calcified amorphous tumor, Right atrium, Rare tumor

## Abstract

**Background:**

Cardiac calcified amorphous tumors (CATs) represent rare, nonneoplastic intraluminal heart masses, with limited case reports in existing literature. Asymptomatic cases localized in the right atrium are particularly unusual.

**Case presentation:**

An asymptomatic 46-year-old male was discovered to have a cardiac mass upon echocardiograph. Echocardiography revealed a 13.2 × 11.8 mm pedunculated mass in the right atrium, attached to the interatrial septum. Then we performed surgical treatment. Histopathology revealed some myocardial tissue, a powdery stained, calcified amorphous area, and a few localized lymphocytes and red blood cells. The final diagnosis confirmed a cardiac CAT.

**Conclusions:**

CATs, rarely occurring endocardium-based pseudotumors, comprise calcium nodules and amorphous fibrin material. Typically presenting as a calcified pedunculated mass, they may arise in any heart chamber, with a significant propensity for distal embolism. Differentiating CATs from calcified atrial myxomas, calcified thrombi, or other cardiac tumors is challenging. Histopathology remains a critical diagnostic cornerstone. Although complete surgical resection is the recommended treatment, anticoagulation and ongoing surveillance may serve as viable alternatives when primary treatment, surgical resection, is deemed excessively hazardous.

## Background

Cardiac calcified amorphous tumors (CATs) are rare benign intraluminal masses, characterized by nodular calcium deposits within fibrin material. Though these tumors can present in any cardiac chamber, they predominantly involve the left ventricle and mitral valve. Owing to their rarity, the exact incidence, pathogenesis, and natural history of CATs remain undefined [1]. Herein, we detail an asymptomatic right atrial CAT case.

## Case presentation

**Fig. 1:**
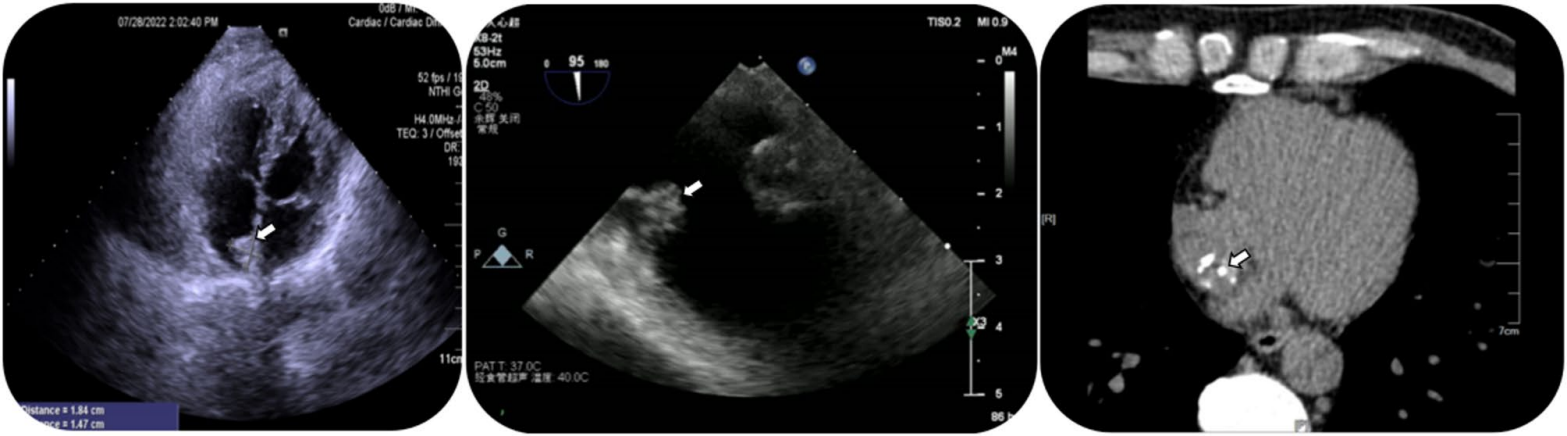
**A**: Two-dimensional echocardiography displays a pedunculated calcified mass in the right atrium. **B**: Transesophageal echocardiography demonstrates a 13.2 × 11.8 mm pedunculated mobile mass attached to the right side of the interatrial septum. **C**: Coronary CTA: intraatrial mass in the right atrium exhibits calcification

**Fig. 2:**
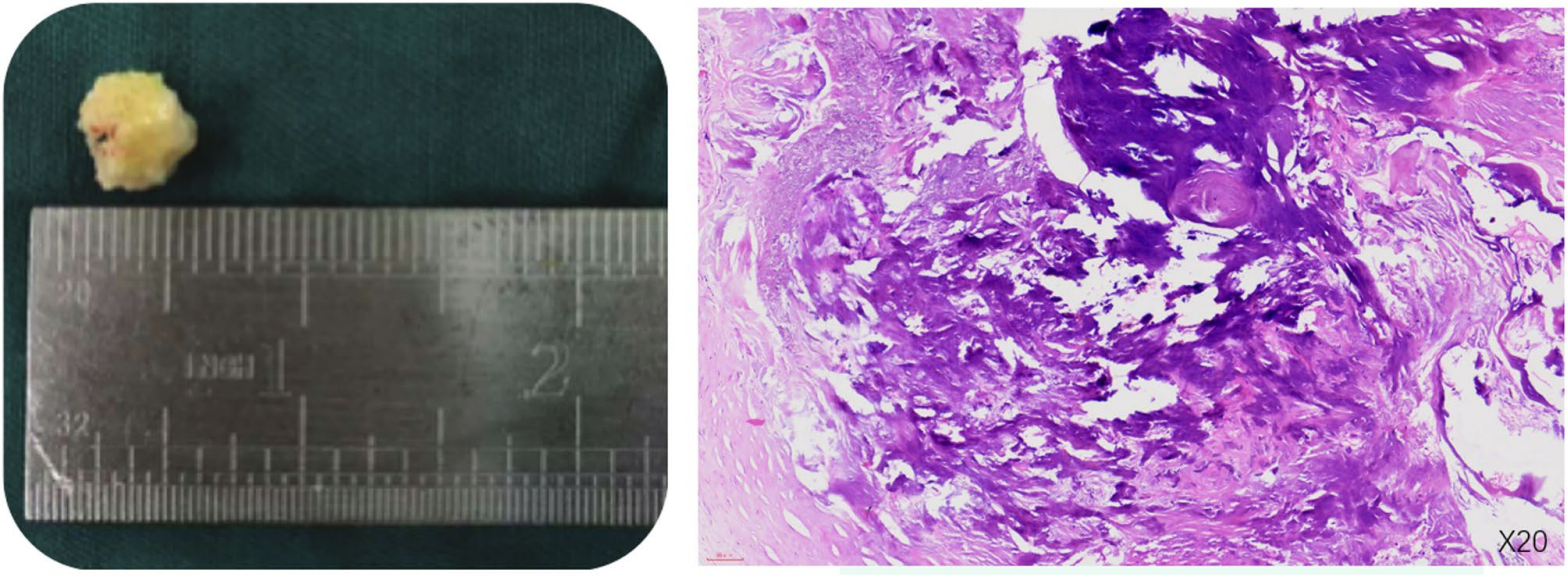
Gross and histological image of the CAT; the mass was removed in an irregular fragment measuring 13 × 13 × 10 mm (**A**); histological examination at 20x magnification (**B**) (Hematoxylin and Eosin staining) demonstrated nodular calcification against an eosinophilic amorphous background; both gross and histological observations were consistent with a cardiac CAT

A 46-year-old male, asymptomatic, presented with a cardiac mass, as detected via echocardiography. The patient reported no chest discomfort or palpitations, with no history of syncope. Previously in good health, the patient’s examination exhibited clear lungs; normal heart sounds; absent murmurs, rubs, or gallops; and no jugular venous distension or peripheral edema. Routine blood tests, including C-reactive protein (CRP), and liver and kidney function were within normal limits. Echocardiographic examination revealed a slightly increased echogenic area near the atrial septum at the top of the right atrium, with clear boundaries, irregular morphology, broad base, uneven internal echo, and limited mobility. Transesophageal ultrasound suggested an abnormal echo attached to the atrial septum on the right atrial surface of the lower part of the atrial septum, with no significant activity, irregular morphology, uneven internal echo, and punctate calcification foci, without any blood flow signal, and the atrial septum was intact and complete. Coronary CTA displayed no significant abnormalities, apart from a calcified intraatrial mass in the right atrium (Fig. 1). Our initial differential diagnosis comprised myxoma with calcification and calcific thrombus. Given the mass’s size, location, and embolic potential, surgical resection was proposed. During surgery, we noted a slightly enlarged right atrium housing a firm, medium-sized tumor (approx. 20 × 20 × 20 mm), with a narrow pedicle (approx. 3 mm wide) attached near the tricuspid valve. The valve structure, subvalvular chordae, and papillary muscles appeared normal. The tumor was completely dissected and removed. Postoperatively, the patient had an uneventful recovery and was discharged one week later. Histopathology revealed some myocardial tissue, a powdery stained, calcified amorphous area, and a few localized lymphocytes and red blood cells, suggesting a CAT. There were no signs of cellular atypia or mitosis (Fig. 2). At a one-year follow-up, the patient remained asymptomatic with no echocardiographic evidence of mass recurrence.

## Discussion and conclusions

Primary cardiac tumors represent a rare clinical phenomenon, with a lifetime incidence of 0.0017-0.02%, with CATs comprising an estimated 2.48% of these primary cardiac tumors [2]. CATs are unusual, endocardium-based intraluminal pseudotumors. They are non-neoplastic masses that consist of calcium nodules and amorphous fibrin material [3]. Reynolds was the first to describe and coin the term “calcified amorphous tumor” in relation to non-neoplastic cardiac masses [4, 5]. Over two decades since the term was first introduced, instances of CATs remain infrequent in literature. A review of available literature revealed only 63 reported cases, affecting individuals between 16 and 85 years old, with a mean age of 58 ± 17 years. The male-to-female ratio was observed to be 24:39. Clinical symptoms typically include dyspnea, syncope, or embolic manifestations that be similar to those associated with other cardiac masses such as cardiac myxomas, thus making diagnosis challenging [6].

A few instances of cardiac CATs were found to be associated with underlying hypercoagulability, atrial fibrillation, trauma, antiphospholipid syndrome, malignancy, and genetic disorders [7]. Cardiac CATs have been correlated with hypertension (16%), valvular heart disease (31%), end-stage renal disease (25%), diabetes (23%), and coronary artery disease (18%), with mitral annular calcification (MAC) being particularly common [3]. Predominant attachment sites were noted to be the mitral valve in 30 cases (48%) and the left ventricle in 16 cases (25%). The most common comorbidity was end-stage renal disease (ESRD), observed in 23 cases (37%). Twenty-one patients (33%) developed embolic symptoms, with both left and right sides of the heart demonstrating an approximately 30% risk of CAT embolism. In the majority of instances (56 cases, 89%), surgical resection was the preferred treatment approach [1, 2].

Despite being benign, a CAT’s size, location, and mobility can lead to recurrent cardiac embolism and blood flow obstruction. Its mobility may contribute to the development of systemic embolism [8]. It has been proposed that CATs could precipitate myocardial infarction either by the CAT itself embolizing from the heart, or via embolization of surface fibrin. A detached CAT typically coexists with a calcified intravascular thrombus. Additionally, thrombosis has been implicated in a few cases of CAT development. Antithrombotic agents may reduce CAT size; daily aspirin (100 mg) administration led to a CAT reduction and disappearance over two months.

To definitively diagnose, symptomatic patients generally require surgical resection and subsequent histopathological examination. Although the optimal treatment for cardiac CAT remains undetermined, complete surgical resection is advocated due to its ability to reduce the risk of complications or embolism and to confirm the diagnosis [9]. Recurrence is rare but may occur primarily due to incomplete surgical removal. Therefore, when first-line therapy (i.e., surgical resection) is deemed too risky, anticoagulation and long-term monitoring may serve as effective alternative treatments [10, 11].

Various histological differential diagnoses of cardiac CAT include myxomas [12], vegetations, echinococcal cysts, and thrombosis. Echocardiography plays an integral role in differentiating the location, mobility, and morphology of a cardiac mass [13]. Kumar’s study characterized CATs on echocardiograms as pedunculated masses predominantly located within the left ventricle, exhibiting diffuse calcifications [14, 15].

Cardiac CAT represents a rare intracardiac nonneoplastic mass, hypothesized to arise from hypercoagulability or abnormal calcium and phosphorus metabolism [16]. Similar to the clinical presentations of other cardiac masses, imaging alone is insufficient for diagnosing CAT. The definitive diagnosis hinges on pathological examination [4, 17].

In conclusion, we report a rare case of a right atrial CAT in a patient with normal renal function. The patient underwent successful atrial mass resection and had a favorable prognosis. However, the precise etiology and treatment strategies for this benign tumor remain uncertain. The infrequency of this tumor’s occurrence in patients with normal renal function and its location in the right atrium underscores the necessity for further investigation into the pathology of this uncommon disease.

## Data Availability

No datasets were generated or analysed during the current study.

## Abbreviations

CATs: Cardiac calcified amorphous tumors
CTA: Coronary computed tomography angiography
CRP: C-reactive protein
MAC: Mitral annular calcification
ESRD: End-stage renal disease
